# Circuit quantum acoustodynamics with surface acoustic waves

**DOI:** 10.1038/s41467-017-01063-9

**Published:** 2017-10-17

**Authors:** Riccardo Manenti, Anton F. Kockum, Andrew Patterson, Tanja Behrle, Joseph Rahamim, Giovanna Tancredi, Franco Nori, Peter J. Leek

**Affiliations:** 1 Clarendon Laboratory, Department of Physics, University of Oxford, OX1 3PU Oxford, UK; 2 Center for Emergent Matter Science, RIKEN, Saitama, 351-0198 Japan; 3 Physics Department, The University of Michigan, Ann Arbor, MI 48109-1040 USA

## Abstract

The experimental investigation of quantum devices incorporating mechanical resonators has opened up new frontiers in the study of quantum mechanics at a macroscopic level. It has recently been shown that surface acoustic waves (SAWs) can be piezoelectrically coupled to superconducting qubits, and confined in high-quality Fabry–Perot cavities in the quantum regime. Here we present measurements of a device in which a superconducting qubit is coupled to a SAW cavity, realising a surface acoustic version of cavity quantum electrodynamics. We use measurements of the AC Stark shift between the two systems to determine the coupling strength, which is in agreement with a theoretical model. This quantum acoustodynamics architecture may be used to develop new quantum acoustic devices in which quantum information is stored in trapped on-chip acoustic wavepackets, and manipulated in ways that are impossible with purely electromagnetic signals, due to the 10^5^ times slower mechanical waves.

## Abstract (web summary)

In this work, Manenti et al. present measurements of a device in which a tuneable transmon qubit is piezoelectrically coupled to a surface acoustic wave cavity, realising circuit quantum acoustodynamic architecture. This may be used to develop new quantum acoustic devices.

## Introduction

The study of the quantum nature of mechanical systems has rapidly increased in the last decade^1–3^. The primary goal of these experiments has been the demonstration of the quantum behaviour of macroscopic objects when suitably isolated from their environment, with the intent to corroborate the validity of quantum mechanics at macroscopic scales. Pioneering work has now experimentally proved the possibility to prepare mechanical objects close to their quantum ground state^4, 5^ and to coherently manipulate their state^6^. These results have encouraged new lines of research utilising mechanical quantum devices, including the development of microwave-optical converters^7^, mechanical quantum memories^8^ and quantum-limited amplifiers^9^, the generation of squeezed vacuum states of mechanical objects^10, 11^, and the detection of non-classical correlations of photon–phonon pairs^12^.

A highly successful architecture for the exchange of single quanta between coupled quantum systems is the solid-state version of cavity quantum electrodynamics (QED), known as circuit QED^13^, in which the electrical interaction between a qubit and a high-quality microwave resonator offers the possibility to reliably control, store, and read out quantum bits of information on a chip. Although many quantum experiments involving mechanical objects have been reported that use an optomechanical coupling between an electric field and a mechanical system^2^, a parallel series of investigations have employed such a circuit-QED type of interaction between mechanical resonators and superconducting qubits^6, 14–16^, which in principle enables full control of the quantum state of the mechanical mode via the qubit.

In contrast to these experiments involving localised mechanical modes, studies have also recently emerged on the coupling of superconducting qubits to travelling surface acoustic waves (SAWs)^17, 18^, which are mechanical perturbations that propagate on the surface of a crystal^19^, and are naturally coupled to superconducting circuits using the piezoelectric effect. As well as being of fundamental interest to study such acoustic waves at the quantum level, they may find uses in quantum signal processing, since their slow speed of travel (five orders of magnitude slower than light) means many-wavelength signals can be manipulated on a mm-scale chip^18, 20, 21^. It has been demonstrated that Fabry–Perot SAW cavities formed using superconducting surface Bragg mirrors can reach quality factors in the 10^5^ range at microwave frequencies^22, 23^, opening up the possibility of realising surface acoustic cavity QED, either with superconducting qubits, or with other solid-state quantum systems^24^. SAW cavities have also been proposed as a potential quantum acousto-optic transducer between superconducting qubits and optical photons by exploiting stimulated Brillouin scattering^25^.

In this work, we present measurements of a device in which a tuneable transmon qubit^26^ is piezoelectrically coupled to a SAW cavity, displaying a surface acoustic version of cavity QED which we call circuit quantum acoustodynamics (QAD). We characterise the dispersive interaction between the two systems in several ways. First, we measure the frequency shift of the acoustic mode as the qubit is flux tuned. Secondly, we measure the acoustic Stark shift of the qubit due to the population of the mechanical resonator and we observe a preferential coupling of the qubit to one longitudinal mode of the acoustic cavity. We extract the coupling and we show that it is in agreement with theoretical expectations. In order to demonstrate the possibility to control the device in the time domain, we show a time-delayed Stark shift made possible by the slow travel of the wave. We also present spectroscopic measurements of the qubit via the Stark shift of the acoustic cavity, indicating that SAWRs can in principle be adopted as an alternative qubit readout scheme in quantum information processors.

## Results

### Description of the experiment

**Fig. 1:**
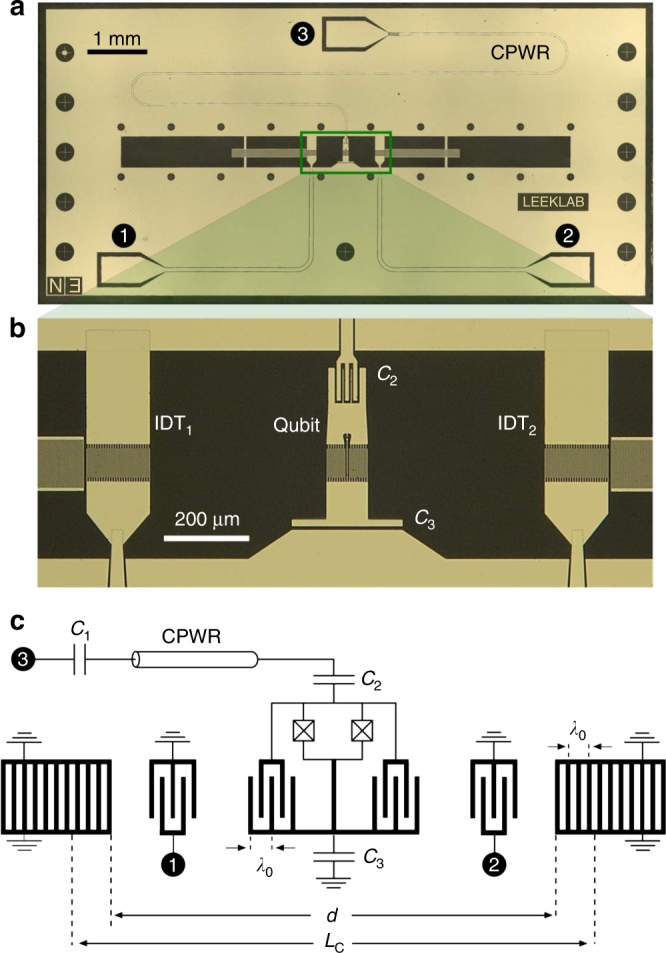
Circuit quantum acoustodynamics device. **a** Optical image of the measured device. In the centre of the chip, a transmon is embedded in a SAW cavity. A coplanar waveguide resonator (CPWR) is coupled to the transmon and measured via port 3. The SAW cavity is probed via two interdigitated transducers (IDTs) connected to ports 1 and 2. **b** Close-up image showing the transmon qubit and SAW IDTs in between the two Bragg gratings that form the SAW cavity. **c** Equivalent electrical circuit of the device incorporating a spatial schematic of the SAW cavity. The geometrical parameters *λ*_0_, *d* and *L*_c_ denote the wavelength, the distance between the two Bragg gratings and the effective length of the cavity, respectively

Our measured device (Fig. 1) is fabricated on ST-X quartz, on which the free SAW speed is *v*_f_ ≈ 3158 m/s at room temperature^19^. This travelling mode is excited by applying an oscillating voltage to the electrodes of an interdigitated transducer (IDT) patterned on the surface of the substrate. The propagating SAW is confined to a small region of the chip between two Bragg mirrors facing each other forming a Fabry–Perot acoustic cavity; each mirror consists of a regular array of shorted metallic strips. A tuneable transmon qubit is situated in the middle of the SAW cavity and consists of a SQUID shunted by an interdigitated capacitance with periodicity *λ*_0_ matching the SAW IDTs (see Supplementary Note 1 for further details on device parameters). The transmon is also coupled to an auxiliary coplanar waveguide resonator (CPWR) employed for independent dispersive qubit readout^27^. All measurements presented hereafter have been performed at the base temperature *T* ≈ 10 mK of a dilution refrigerator. Microwave ports 1 and 2 are connected to room temperature via low (≈16 dB) attenuation lines in order to easily populate and measure the SAW cavity modes, while port 3 is highly attenuated (≈70 dB) such that the CPWR and qubit are close to their quantum-mechanical ground states.

### SAW cavity response in frequency and time domain

**Fig. 2:**
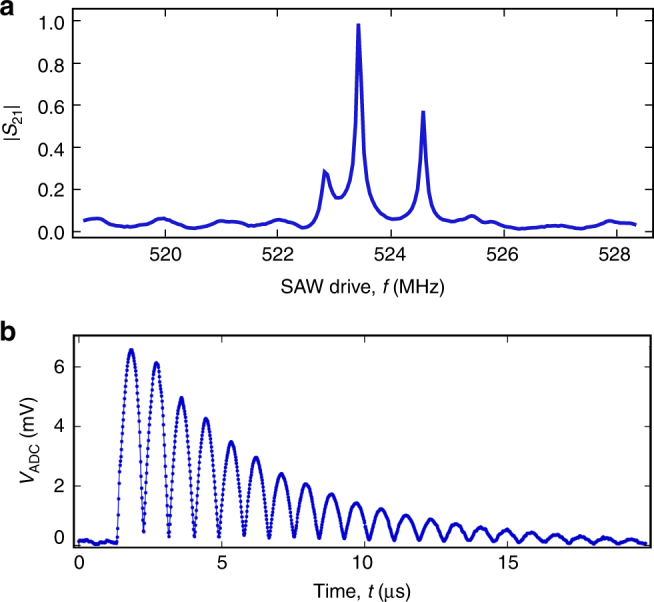
SAW cavity response. **a** Normalised linear magnitude of the measured transmission coefficient *S*_21_ of the 2-port SAWR (blue solid line). The transmitted signal has been acquired with a vector network analyser with input power set at −30 dBm. **b** Time resolved measurement of the 2-port SAWR. This measurement has been performed by applying a 800 ns ≲ 2*L*_c_/*v*_e_ electrical pulse to IDT_1_ and acquiring the output signal from IDT_2_. The graph shows the voltage difference at the input of the acquisition card

The SAW cavity contains two transducers for the excitation and detection of the acoustic wave. Figure 2a shows the transmission coefficient *S*_21_ of the cavity, measured via the two IDTs. The frequency of the central mechanical mode is *f*_m2_ = 523.435 MHz, while side peaks seen at *f*_m1_ = 522.83 MHz and *f*_m3_ = 524.58 MHz are likely to be additional mechanical modes. The quality factors of these modes, *Q*_m1,m2,m3_ = {4820, 6980, 7580}, are obtained from additional measurements of *S*_11_ (see Supplementary Note 1 for more information). Since the periodicity of the IDTs is set to *λ*_0_ = 6 in fabrication, the central mode frequency is consistent with a speed of sound of *v*_e_ = *f*_m2_ *λ*_0_ = 3140.6 m/s, assuming a symmetric device. The slight difference between *v*_e_ and the textbook room temperature value *v*_f_ may be due to slight device asymmetry and/or stiffness tensor changes or crystal contraction at millikelvin temperatures. Note that the SAW cavity modes are not in their ground state, due to the mode frequencies *f*_m*i*_ ≲ *k*_B_ *T*/*h* and low-attenuation connections to room temperature.

The acoustic nature of the observed resonant modes can be further tested by measuring the device response to a short coherent drive pulse (Fig. 2b). An exponentially decaying train of pulses is clearly observed in the response, consistent with the applied 800 ns pulse reflecting back and forth between the mirrors of the cavity. The decay time of the pulses, the lifetime of phonons in the SAW cavity, is *τ* ≈ 2.4 μs. The pulses measured in the response are separated by Δ*t* = 870 ns, consistent with a cavity length of *L*_c_ = *v*_e_Δ*t*/2 ≈ 1365 μm. As expected, the cavity length is slightly longer than the distance between the two gratings *d* = 1260 μm. This is consistent with the fact that SAWs slightly penetrate into the gratings by an amount *L*_p_ = (*L*_c_ − *d*)/2 = 55 μm before being efficiently reflected. The frequency spacing of adjacent modes (the free spectral range) of the cavity are also consistent with the same cavity length, ${L_{\rm{c}}} = {v_{\rm{e}}}{\rm{/}}2\left| {{f_{{\rm{m2}}}} - {f_{{\rm{m3}}}}} \right| \approx 1365$ μm, where we have used the two higher-quality modes *f*_m2_ and *f*_m3_. Note, however, that *f*_m1_ is slightly closer in frequency to *f*_m2_. This asymmetric behaviour in the frequency domain may be due to the fact that the grating stopband does not perfectly coincide with the resonant frequency of the IDTs^28^.

### Interaction between a SAW cavity and a superconducting qubit

Having characterised the SAW cavity, we now proceed to examine its interaction with the superconducting qubit, a flux-tuneable transmon. An appropriate quantum-mechanical description of this system (including the readout CPWR) is the generalised Jaynes–Cummings Hamiltonian of two resonators both coupled to the same transmon qubit (for reasons that will become clear, we will only consider the central acoustic mode at *f*_m2_, which we hereafter refer to as the SAW resonator, SAWR):

1 $$\begin{array}{*{20}{l}}\\ {\hat H{\rm{/}}h} \hfill & = \hfill & {\mathop {\sum}\limits_j {f_j}(\Phi )\left| j \right\rangle \left\langle j \right| + {f_{\rm{r}}}{{\hat a}^\dag }\hat a + {f_{{\rm{m2}}}}{{\hat b}^\dag }\hat b } \hfill \\ \\ {} \hfill & {} \hfill & { + \mathop {\sum}\limits_{i,j} \left[ {{g_{ij}}(\Phi )\left| i \right\rangle \left\langle j \right|\left( {\hat a + {{\hat a}^\dag }} \right) + {\lambda _{{\rm{m2}},ij}}(\Phi )\left| i \right\rangle \left\langle j \right|\left( {\hat b + {{\hat b}^\dag }} \right)} \right],} \hfill \\ \end{array}$$

where *f*_*j*_(Φ) are the flux dependent transmon transition frequencies, *f*_r_ = 5.83 GHz is the CPWR frequency, $\hat a$ $\left( {{{\hat a}^\dag }} \right)$ and $\hat b$ $( {{{\hat b}^\dag }} )$ are the annihilation (creation) operators of the microwave cavity and of the mechanical resonator, respectively and *g*_*ij*_(Φ) [*λ*_m2,*ij*_(Φ)] is the coupling strength between the qubit and the CPWR (SAWR). Hereafter, we will denote the coupling strength between the CPWR (SAWR) and the first energy level of the qubit simply by *g* (*λ*_m2_). The transition frequency *f*_q_ between the first two energy levels of the qubit is tuned by applying an external magnetic flux Φ to its superconducting loop, and its value is given by:

2 $$h{f_{\rm{q}}}(\Phi ) = \sqrt {8{E_{\rm{C}}}{E_{{\rm{J0}}}}{\rm{cos}}\left| {\pi \Phi {\rm{/}}{\Phi _0}} \right|} - {E_{\rm{C}}},$$

where Φ_0_ = *h*/2*e* is the magnetic flux quantum, and *E*_C_ = *h* × 0.31 GHz, *E*_J0_ = *h* × 10.7 GHz are the Coulomb and maximum Josephson energy of the qubit (obtained from standard qubit spectroscopy). The electrical coupling between the CPWR and the qubit mainly originates from the capacitance *C*_2_ shown in Fig. 1b. The acoustic coupling is instead due to the potential difference generated by the acoustic wave on the electrodes of the qubit and is given by:

3 $${\lambda _{{\rm{m2}}}}\left( {\Phi ,f} \right) = \frac{e}{h}\frac{{{C_{\rm{q}}}}}{{{C_\Sigma }}}{\left( {\frac{{{E_{\rm{J}}}(\Phi )}}{{2{E_{\rm{C}}}}}} \right)^{\!\!\!1/4}}\frac{{{e_{{\rm{pz}}}}}}{\varepsilon }\sqrt {\frac{\hbar }{{2\rho {A_{\rm{c}}}{v_{\rm{e}}}}}} A(f),$$

where *e* is the electron charge, *C*_q_ and *C*_Σ_ are the qubit capacitance and the total capacitance seen by the qubit respectively, *e*_pz_ is the piezoelectric coupling coefficient, *ε* is the substrate permittivity, *ρ* is the substrate mass density, *A*_c_ is the acoustic cavity area and *A*(*f*) is a normalised array factor (see Supplementary Note 2 for a derivation of Eq. (3)). From values related to our experiment, this formula predicts *λ*_m2_ = 6.0 MHz (for a qubit frequency of *f*_q_ = 2.52 GHz, for comparison with later measurements).

### SAW cavity response as a function of flux

**Fig. 3:**
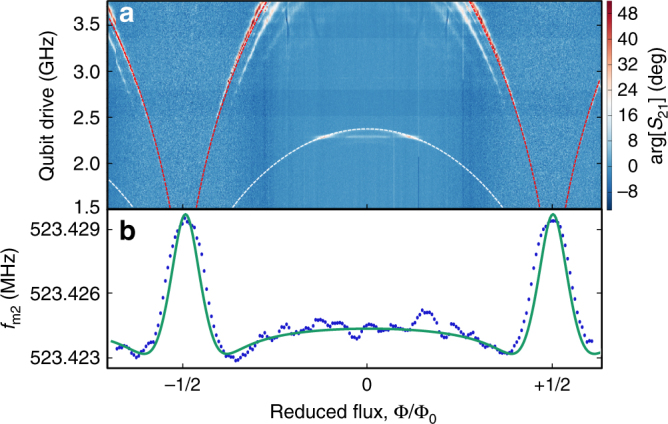
Flux dependent acoustic shift. **a** Qubit spectroscopy performed with the CPWR as a function of reduced magnetic flux. The red dashed line is a fit to Eq. (2) and indicates the qubit transition frequency *f*_q_(Φ). The white dashed line, with analytical form *f*_q_(Φ)/2, denotes an excitation of the qubit via a two photon process. **b** Measured resonant frequency of the acoustic mode *f*_m2_ as a function of reduced flux at fixed SAW drive power of −80 dBm (blue points) and numerical model based on Eq. (1) (green solid curve)

As a first probe of the interaction between the qubit and the SAW mode, we measure both the frequency of the qubit (via the CPWR) and the acoustic mode *f*_m2_ as a function of magnetic flux Φ (Fig. 3). The qubit frequency (Fig. 3a) fits well to Eq. (2), while the SAW mode frequency (Fig. 3b) also shows a flux dependence with the same periodicity. By fitting the experimental curve of Fig. 3b with a QuTiP numerical model^29^ based on Eq. (1), we can extract the value of the acoustic coupling and we find *λ*_m2_ = 5.7 ± 0.5 MHz at *f*_q_ = 2.52 GHz. In this model, the transmon coupling strengths and level spacings are calculated by diagonalising the Hamiltonian for a Cooper-pair box including many charge states^26^. The free parameters of the model are the coupling strength *λ*_m2_, the asymmetry of the critical currents of the two Josephson junctions [*d*_sym_ = (*I*_c1_ − *I*_c2_)/(*I*_c1_ + *I*_c2_) = 0.09] and the effective temperature of the device (*T* = 85 mK). The additional SAW modes at *f*_m1_ and *f*_m3_ do not show any detectable flux dependence. This is in agreement with the expectation that these modes are antisymmetric with respect to the centre of the cavity, whereas the central mode *f*_m2_ and the qubit transducer geometry are both symmetric.

### Acoustic Stark shift of the qubit

**Fig. 4:**
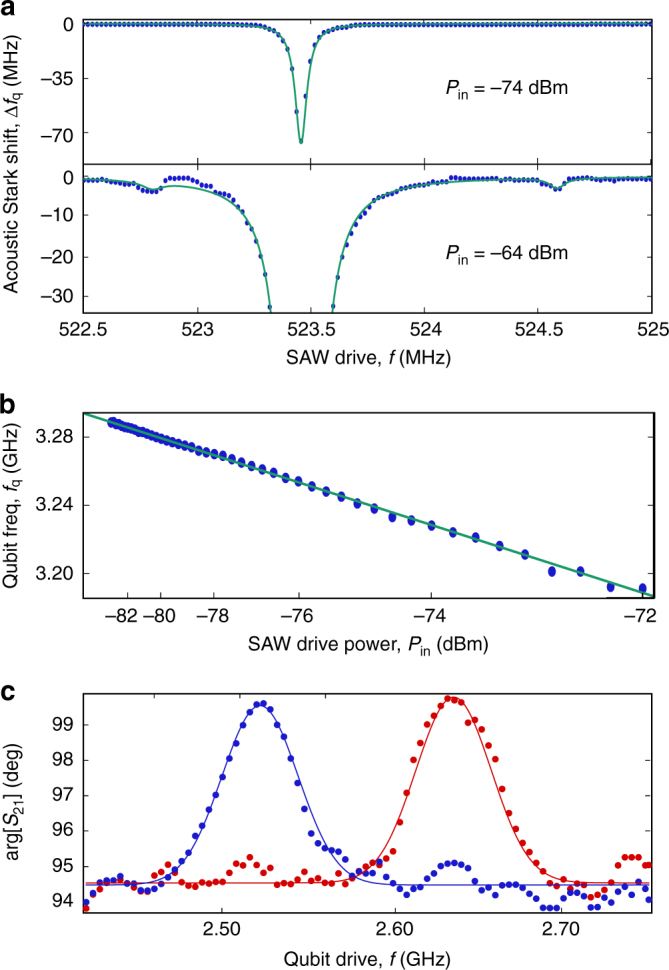
Acoustic Stark shift. **a** Qubit frequency shift as a function of SAW drive at two different powers: −74 dBm (top panel) and −64 dBm (bottom panel). The green curves are Lorentzian fits to the data points. **b** Qubit frequency as a function of SAW drive power *P*_in_ at a fixed SAW frequency *f*_m2_ (blue points). The green solid line is a linear fit to the data. **c** Qubit spectroscopy performed with the SAWR at two different flux values [0.395Φ_0_ (red points) and 0.403Φ_0_ (blue points)]. The solid lines are gaussian fits to the data points

A second method to investigate the acoustic coupling between the qubit and the SAW is to measure the AC Stark shift between the two systems in the far-detuned limit (Fig. 4), in which the Hamiltonian of the SAW-qubit system becomes:

4 $${\hat H_{{\rm{disp}}}}{\rm{/}}h \approx {f_{{\rm{m2}}}}{\hat b^\dag }\hat b + \frac{1}{2}\left( {{f_{\rm{q}}} + 2\chi {{\hat b}^\dag }\hat b + \chi } \right){\hat \sigma _z},$$

where ${\hat \sigma _z}$ is a Pauli operator and we have approximated the transmon as a two-level system for simplicity. We first set the magnetic flux such that the qubit frequency is *f*_q_ = 3.29 GHz, and the qubit–SAW detuning is $\Delta = 2.77\,{\rm{GHz}} \gg {\lambda _{{\rm{m2}}}}$. In Fig. 4a, we show the qubit frequency shift as a function of SAW drive frequency close to the acoustic modes at two different drive powers. At the lower power of *P*_in_ = −74 dBm, we clearly observe a qubit frequency shift only at the frequency of the central SAW mode *f*_m2_. The shift fits well to a Lorentzian centred at *f*_m2_, and has a FWHM of 60 ± 5 kHz, close to that obtained for the SAW mode measured via *S*_21_. No such frequency shift is observed at the other SAW mode frequencies *f*_m1_ and *f*_m3_ until higher power. From a second measurement at *P*_in_ = −64 dBm, high enough to observe small shifts at *f*_m1_ and *f*_m3_, we can estimate the coupling of the qubit to these additional modes. Assuming that *λ*_m2_ = 5.7 MHz from the fit to the flux dependence of *f*_m2_, and taking into account the different drive powers of the two experiments, we can estimate the coupling to the two side modes to be *λ*_m1_ ≈ 380 kHz and *λ*_m3_ ≈ 340 kHz, more than an order of magnitude lower than the coupling to the central mode. Figure 4b illustrates the qubit frequency shift as a function of drive power at frequency *f*_m2_. The shift is observed to be linear, in agreement with the AC Stark effect.

### Acoustic dispersive readout of the qubit

In a complementary experiment, we measure the Stark shift of the SAW mode frequency when the qubit excited state is populated (Fig. 4c), an acoustic equivalent of circuit QED dispersive qubit readout^27^. This measurement can be used to extract an independent estimate of the acoustic coupling *λ*_m2_. We carry out such a measurement at two different values of the magnetic flux, in both cases measuring the phase shift Δ*ϕ* of a probe drive at *f*_m2_ under strong drive of the qubit. We have $\Delta \phi = 2\,{\rm{arctan}}\left( {\left| \chi \right|{\rm{/}}{\kappa _{{\rm{m2}}}}} \right)$, where *κ*_m2_ is the mechanical mode linewidth, $\chi = - \lambda _{{\rm{m2}}}^2{E_{\rm{C}}}{\rm{/}}\Delta \left( {\Delta - {E_{\rm{C}}}} \right)$ is the dispersive shift for a transmon and Δ = *f*_q_ − *f*_m2_ is the frequency detuning. From Fig. 4c, we have Δ = 2.00 GHz and Δ*ϕ* = 4.9° ± 0.3° and since *κ*_m2_ = 75 kHz, we find $\left| \chi \right|$ = 3.2 kHz and *λ*_m2_ = 5.9 ± 0.2 MHz, in close agreement with our earlier estimate obtained by fitting the flux dependence of *f*_m2_. We can now use this measurement of *χ* to obtain an estimate for the average coherent phonon population $\bar n = \langle {{{\hat b}^\dag }\hat b} \rangle$ of the SAW mode in the experiment shown in Fig. 4a, obtaining $\bar n \approx {10^4}\left( {{{10}^5}} \right)$ for *P*_in_ = −74(−64) dBm. These values are within the limit in which the dispersive Hamiltonian remains valid^30^, ${\lambda _{{\rm{m2}}}}\sqrt {\bar n} < \Delta$.

### Time-delayed acoustic Stark shift of the qubit

**Fig. 5:**
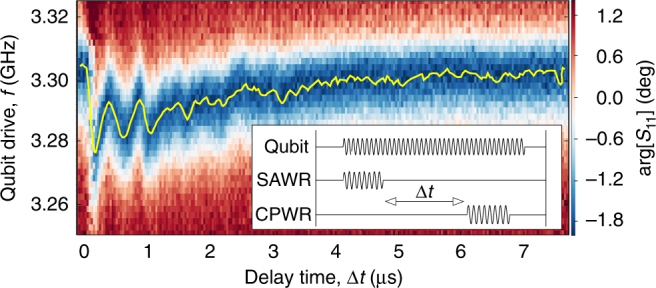
Time-delayed acoustic Stark shift. Measured time-delayed acoustic Stark shift of the qubit. The yellow solid line indicates the qubit frequency. Inset: pulse scheme related to this experiment: a continuous drive excites the qubit, while two short 100 ns pulses delayed by Δ*t* drive the SAWR and the CPWR for the readout

We finally report an experiment in which we use the slow travel of the acoustic wave to apply a time-delayed Stark shift to the qubit, occurring as a SAW pulse passes the qubit inside the SAW cavity (Fig. 5). A short 100 ns pulse is first applied to one SAW IDT, then a time-delayed pulsed measurement of the qubit is subsequently carried out via the CPWR (with a 100 ns measurement pulse). A continuous drive is applied to the qubit throughout the experiment, the frequency of which is varied to determine the qubit frequency. The qubit is observed to shift lower in frequency initially at a time 170 ns after the SAW pulse is applied, exactly consistent with the time-of-flight of the SAW pulse between the IDT and qubit. Several further frequency dips are then observed, spaced by ∼430 ns, again consistent with the SAW time-of-flight from qubit to one Bragg mirror and back again. The qubit frequency is observed to decay back to its undisturbed value over a timescale of ≈3 μs, similar to the phonon lifetime of the SAW cavity. The multiple reflections between qubit, IDTs and Bragg mirrors likely serve to smooth out the response beyond a time delay of around 1 μs. As well as demonstrating the unique slow propagation of SAWs in a quantum device, this experiment also serves to further prove that the Stark shifts that we observe are indeed due to the acoustic field of the SAW mode, rather than a crosstalk of the electromagnetic signal applied to the IDT directly to the qubit.

### Superconducting qubit coherence

Although, as detailed above, we have been able to observe clear signatures of the coherent coupling between a transmon and SAW resonator in our device, the coherence of our transmon (*T*_1(2)_ = 46(67) ns at *f*_q_ = 2.6 GHz (see Supplementary Note 1 for more details)) is significantly lower than the current state of the art. There are several likely explanations for this short coherence. Firstly, the low-temperature dielectric loss tangent of our quartz substrate may be significantly higher than the low-loss sapphire and silicon substrates typically used in current superconducting circuits. Secondly, our transmon geometry necessarily includes a fine-pitched interdigitated capacitor to couple to the SAWs, which increases the dielectric participation ratio with respect to current high-coherence designs^31^. Lastly, since quartz is a piezoelectric material, there may be a noticeable contribution to qubit energy relaxation from bulk (as opposed to surface) phonon emission.

## Discussion

The prototype quantum acoustic device that we have presented here may be improved, opening up the possibility of using cavity-trapped SAWs for quantum memories, time delays and quantum signal filtering applications. In particular, we have used a relatively weak piezoelectric substrate for our experiment, nevertheless achieving a qubit–SAWR coupling strength of 5.7 MHz. Stronger piezoelectrics such as lithium niobate or zinc oxide could dramatically increase this coupling strength. This could have the additional benefit of enabling the qubit coherence to be improved, as the electric field of the qubit could be designed to only partially rather than fully reside in the piezoelectric substrate (which in the present case likely limits coherence due to undesired bulk acoustic emission). The 10^5^ times reduced speed of travel of SAWs compared to electromagnetic signals also makes our device a miniaturised mechanical implementation of traditional cavity QED, and an ideal engineered platform to push the boundaries of cavity QED physics, opening up the possibility to explore, for instance, strong coupling multimode cavity QED with mechanical devices.

### Data availablity

The data that support the findings of this study are available from the corresponding author upon reasonable request.

## Electronic supplementary material

Supplementary Information
