# Supplementary material for: Circuit quantum acoustodynamics with surface acoustic waves
Source: Nat Commun. 2017 Oct 17;8:975. doi: 10.1038/s41467-017-01063-9 (PMC5715021; doi:10.1038/s41467-017-01063-9)
Supplement: Supplementary file 1 — Supplementary Information [file 41467_2017_1063_MOESM1_ESM.pdf]

Supplementary Note 1: Device parameters

In Supplementary Fig. 1, we present a schematic of a tuneable transmon embedded in a 2-port SAW resonator. The acoustic resonator consists of two interdigitated transducers (IDTs) and two gratings. Each IDT is an interdigitated capacitance with $N_\mathrm{t} = 51$ fingers. The periodicity of the transducers is $\lambda_0 = 6\,\mu\mathrm{m}$ and their horizontal length is $L_\mathrm{t} = (2N_\mathrm{t} - 1)\lambda_0/4 = 151.5\,\mu\mathrm{m}$. One bus bar of the IDT is grounded whereas the opposite one is connected to a waveguide. As regards the gratings, they consist of $N_\mathrm{g} = 400$ shorted metallic strips connected to ground with a periodicity of $\lambda_0/2$. The horizontal length of each grating is $L_\mathrm{g} = (2N_\mathrm{g} - 1)\lambda_0/4 = 1198.5\,\mu\mathrm{m}$. In the remainder, we will call the left and right transducers IDT_1 and IDT_2 respectively. The same nomenclature is adopted for the gratings.

For optimal reflection, the minimal distance between IDT_1 and $\mathrm{grating}_1$ must satisfy the relation $d_1 = (n_1/2 - 1/4)\lambda_0 - \lambda_0/8$, where $n_1 \in \mathbb{N}^+$. To minimise the cavity length we opted for $n_1 = 3$, leading to $d_1 = 6.75\,\mu\mathrm{m}$ (a lower value of n_1 might have caused some fabrication issues). The distance d_4 between IDT_2 and $\mathrm{grating}_2$ is the same as d_1. Finally, the minimal distance between the two IDTs satisfies the relation $\Delta_0 = (n_2 - 3/4)\lambda_0$ where $n_2 \in \mathbb{N}^+$. The value of n_2 can be chosen based on some considerations. From previous experiments[1], it has been noted that the internal quality factor of a SAW resonator increases as the distance between the two gratings increases. It has also been observed that the number of longitudinal modes supported by the acoustic cavity increases with increasing distance between the two gratings. Lastly, the coupling between the qubit and the SAWR is inversely proportional to the square root of the acoustic cavity

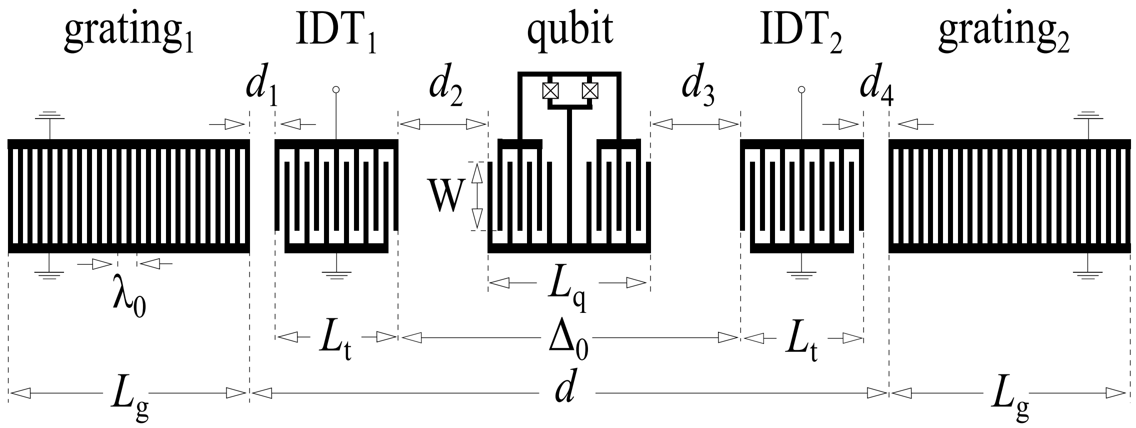

Supplementary Figure 1 | Schematic of a transmon qubit embedded in a 2-port SAW resonator. The 2-port SAW resonator consists of two IDTs and two gratings. The tuneable qubit is formed by a SQUID shunted by an interdigitated capacitance. Due to limited space, the device represented in this figure is not in scale and the number of fingers in the qubit, IDTs and gratings has been reduced with respect to the real device (see text for further details).

area (see Supplementary Note 2). For a better coherence, we aimed at having an internal quality factor as high as possible; at the same time, we wanted to maximise the acoustic coupling and we wanted our cavity to support a small number of longitudinal modes. For these reasons, we have set n_2 to the reasonable value of 158 and therefore $\Delta_0 = 943.5\,\mu\text{m}$. As explained in the main text, our acoustic cavity supports three longitudinal modes separated by about $1\,\text{MHz}$.

The tuneable transmon is placed in the middle of the acoustic cavity. It consists of a $4 \times 4.57\,\mu\text{m}^2$ SQUID shunted by an interdigitated capacitance. The capacitance of the qubit resembles a transducer itself: it consists of $N_\text{q} = 30$ fingers with one additional central finger connecting the two electrodes of the capacitance (see Supplementary Fig. 1). The interdigitated structure of the qubit capacitance has the same periodicity as the IDTs. Unlike these acoustic components, the qubit is connected neither to ground nor to any other waveguide (it is only capacitively coupled to

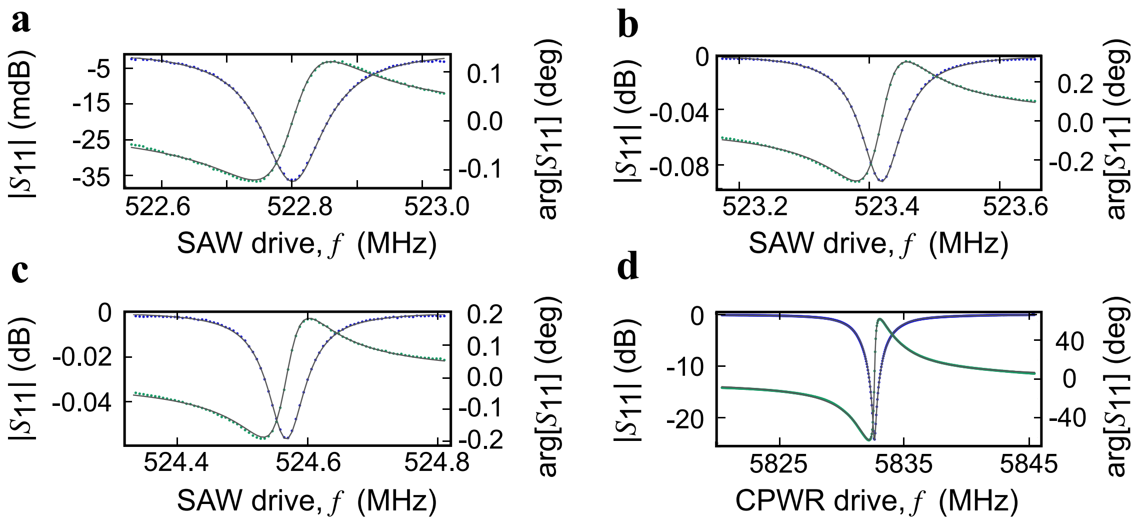

Supplementary Figure 2 | Reflection coefficient of the 2-port SAWR and CPWR. a-c, Magnitude (blue) and phase (green) of the measured reflection coefficient $S_{11}(f)$ of the longitudinal modes f_{m1}, f_{m2} and f_{m3}. Solid lines are a fit to equation (1). **d**, Magnitude (blue) and phase (green) of the measured reflection coefficient $S_{11}(f)$ of the CPWR mode f_{r}. Solid lines are a fit to equation (1). A background due to the measurement setup has been subtracted in all these frequency responses.

ground and to a CPWR not shown in the figure).

The length of the fingers in the IDTs and qubit is denoted by W. A large value of W would considerably decrease diffraction losses[1]; at the same time, a small value of W would decrease the acoustic cavity area and increase the acoustic coupling (see Supplementary Note 2). A tradeoff between these two limits lead us to $W = 11.66\lambda_0 = 70\,\mu\mathrm{m}$. Note that at low temperatures, the device undergoes a non-isotropic contraction and the distances between, as well as the dimensions of the acoustic components may vary on the order of tens of nanometers.

By applying an oscillating voltage to one IDT, it is possible to generate a surface acoustic

wave. The frequency of the wave is given by the simple formula $f = v_\text{e}/\lambda$, where v_e is an effective speed of sound. An optimal transduction is achieved when $\lambda = \lambda_0$, which leads to the central resonant frequency of the device $f_0 = v_\text{e}/\lambda_0$. Assuming that f_m2 is the central resonance, we can extract an effective speed of sound of $v_\text{e} = \lambda_0 \times f_\text{m2} = 3140.6\,\text{m/s}$. The acoustic wave is eventually collected by the second transducer generating a potential difference on its electrodes. In our setup, we used a vector network analyzer (VNA) to acquire the transmitted signal from IDT_1 to IDT_2; the results of this measurement are shown in Fig. 2a of the main text. The SAWR can also be measured in reflection. In Supplementary Fig. 2a-c we present the measured reflection coefficient $S_{11}(f)$ of the 2-port SAW resonator around the three longitudinal modes f_m1, f_m2 and f_m3. Close to resonance, the SAWR can be modelled with an RLC equivalent circuit. According to this model, the analytical expression of the reflection coefficient takes the form:

$$S_{11}\left(f\right) = \frac{\left(Q_\text{m,e} - Q_\text{m,i}\right)/Q_\text{m,e} + 2iQ_\text{m,i}\left(f - f_\text{m}\right)/f}{\left(Q_\text{m,e} + Q_\text{m,i}\right)/Q_\text{m,e} + 2iQ_\text{m,i}\left(f - f_\text{m}\right)/f}. \quad (1)$$

Here $Q_\text{m,i}$ is the internal quality factor of the mechanical mode and $Q_\text{m,e}$ is the external quality factor due to the presence of the IDT and measurement port. From a fit to the experimental data, we find $Q_\text{m1,i} = 4830$, $Q_\text{m2,i} = 7020$, $Q_\text{m3,i} = 7600$, $Q_\text{m1,e} = 2.34 \times 10^6$, $Q_\text{m2,e} = 1.33 \times 10^6$, and $Q_\text{m3,e} = 2.33 \times 10^6$. In terms of loss rate, these values can be expressed as $\kappa_\text{m1} = 108\,\text{kHz}$, $\kappa_\text{m2} = 75\,\text{kHz}$ and $\kappa_\text{m3} = 69\,\text{kHz}$.

For completeness, we also report the measured reflection coefficient of the CPWR in Supplementary Fig. 2d. Its resonant frequency depends on its length L_r and the effective dielectric constant of the substrate ε_eff in the following way: $f_\text{r} = c/2L_\text{r}\sqrt{\varepsilon_\text{eff}}$, where $L_\text{r} = 14\,100\,\mu\text{m}$, and $\varepsilon_\text{eff} \approx 3.3$ for quartz. Finally, by fitting the CPWR response in the frequency domain, we ob-

tain $Q_{\mathrm{m2,i}} = 3240$ and $Q_{\mathrm{m3,e}} = 3630$. In terms of loss rate, these values can be expressed as $\kappa_{\mathrm{r}} = 3.41\,\mathrm{MHz}$.

When the wave bounces against the mirrors, it slightly penetrates into this regular array of metallic fingers. The distance that the wave travels into this periodic structure is called penetration depth L_{p}. The longitudinal cavity length is given by the sum of the distance between the two gratings and the penetration depth into them: $L_{\mathrm{c}} = d+2L_{\mathrm{p}}$. From the frequency difference between f_{m3} and f_{m2}, we can extract the cavity length: $L_{\mathrm{c}} = v_{\mathrm{e}}/2|f_{\mathrm{m3}} - f_{\mathrm{m2}}| = 1370\,\mu\mathrm{m}$. As explained in the main text, this value is in agreement with measurements performed in the time domain. From the value of the cavity length, we can derive the penetration depth $L_{\mathrm{p}} = 55\,\mu\mathrm{m}$. The reflectivity $|r_s|$ of each finger in the grating can be easily obtained from the relation $4|r_s|L_{\mathrm{p}} = \lambda_0 \tanh(|r_s|N_{\mathrm{g}})$, whence $r_s = |0.0273|$. All of the parameters presented so far are listed in Supplementary Table 1.

We conclude this section presenting some observations concerning the coherence time of our qubit. In order to extract the decay time T_1 of our transmon, we performed an inversion recovery experiment. The pulse scheme used to extract T_1 is shown in the inset of Supplementary Fig. 3a: a π pulse is applied to the qubit followed by a readout pulse at the CPWR frequency. Supplementary Fig. 3a shows the exponential decay of the qubit population as a function of the delay between the two pulses. The data points fit well to the exponential model:

$$P_{\mathrm{e}}(t) = \frac{1 + \langle \hat{\sigma}_z(t) \rangle}{2} = A + B\exp\left(-\Delta t/T_1\right), \tag{2}$$

where $A = 32.878\,\mathrm{mV}$ is an offset, $B = 0.3496\,\mathrm{mV}$ is a scaling factor and $T_1 = 46\,\mathrm{ns}$ is the decay time of the qubit (this experiment has been performed with the qubit frequency fixed at

Qubit	Coulomb energy	$E_\mathrm{C} = 0.310\,\mathrm{GHz}$
	Maximum Josephson energy	$E_\mathrm{J0} = 10.704\,\mathrm{GHz}$
	$E_\mathrm{J0}/E_\mathrm{C}$	34.5
	Relaxation time	$T_1 = 46\,\mathrm{ns}$ (at $f_\mathrm{q} = 2.6\,\mathrm{GHz}$)
	Dephasing time	$T_2 = 67\,\mathrm{ns}$ (at $f_\mathrm{q} = 2.6\,\mathrm{GHz}$)
	Qubit frequency	$f_\mathrm{q}(\Phi) = \left(\sqrt{8E_\mathrm{C}E_\mathrm{J0}\cos\lvert\pi\Phi/\Phi_0\rvert} - E_\mathrm{C}\right)/h$
CPWR	Resonant frequency and linewidth	$f_\mathrm{r} = 5.83\,\mathrm{GHz}$ $\kappa_\mathrm{r} = 3.41\,\mathrm{MHz}$
	Internal and external quality factor	$Q_\mathrm{r,i} = 3240$ $Q_\mathrm{r,e} = 3630$
	Qubit - CPWR coupling strength	$g = 69\,\mathrm{MHz}$
SAWR	Resonant frequencies and linewidths	$f_\mathrm{m1} = 522.825\,\mathrm{MHz}$ $\kappa_\mathrm{m1} = 108\,\mathrm{kHz}$
	(in reflection)	$f_\mathrm{m2} = 523.426\,\mathrm{MHz}$ $\kappa_\mathrm{m2} = 75\,\mathrm{kHz}$
		$f_\mathrm{m3} = 524.575\,\mathrm{MHz}$ $\kappa_\mathrm{m3} = 69\,\mathrm{kHz}$
	Internal/external quality factors	$Q_\mathrm{m1,i} = 4830$ $Q_\mathrm{m1,e} = 2.34 \times 10^6$
	(in reflection)	$Q_\mathrm{m2,i} = 7020$ $Q_\mathrm{m2,e} = 1.33 \times 10^6$
		$Q_\mathrm{m3,i} = 7600$ $Q_\mathrm{m3,e} = 2.33 \times 10^6$
	Periodicity	$\lambda_0 = 6\,\mu\mathrm{m}$
	Effective speed of sound	$v_\mathrm{e} = 3140.6\,\mathrm{m/s}$
	Fingers in each IDT	$N_\mathrm{t} = 51$
	Fingers in each grating	$N_\mathrm{g} = 400$
	Fingers in the qubit capacitance	$N_\mathrm{q} = 31$
	Length of each finger	$W = 70\,\mu\mathrm{m} = 11.66\lambda_0$
	Distance between grating and IDT	$d_1 = (n_1/2 - 1/4)\lambda_0 - \lambda_0/8\rvert_{n_1=3} = 6.75\,\mu\mathrm{m}$
	Distance between gratings	$d = 1260\,\mu\mathrm{m} = v_\mathrm{e} \times 401\,\mathrm{ns} =$
	Distance between centre of IDTs	$d_\mathrm{IDT} = 1100\,\mu\mathrm{m}$
	Cavity length	$L_\mathrm{c} = 1365\,\mu\mathrm{m}$
	Cavity area	$A = W \times L_\mathrm{c} = 95\,900\,\mu\mathrm{m}^2$
	Qubit - SAWR coupling strength	$\lambda_\mathrm{m2}(f_\mathrm{q} = 2.5\,\mathrm{GHz}) = 5.7\,\mathrm{MHz}$

Supplementary Table 1 | Device parameters.

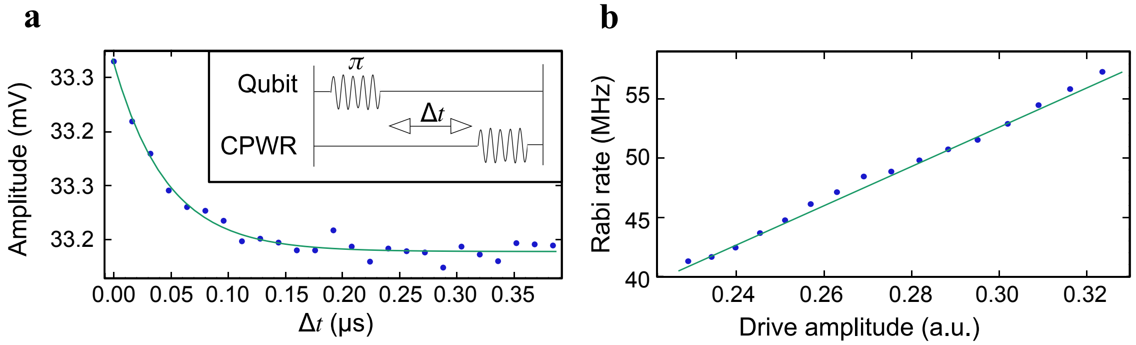

Supplementary Figure 3 | Qubit coherence. a, Inversion recovery experiment to extract the qubit decay time T_1. The solid green curve based on equation (2) fits well the data points (blue dots). **b**, Rabi rate for different values of drive amplitude (blue points). As expected, the data points fit well to a linear dependence (green solid line).

$f_\mathrm{q} = 2.9\,\mathrm{GHz}$). We have also performed Rabi oscillations of the qubit using the CPWR as readout. Supplementary Fig. 3b shows the frequency of Rabi oscillations as a function of drive amplitude: as expected, there is a linear dependence between these two variables.

Supplementary Note 2: Coupling strength between a charge qubit and a SAW cavity

When a surface perturbation on a piezoelectric crystal travels through an interdigitated capacitor with the same periodicity as the incoming wave, the capacitor develops an oscillating voltage on its electrodes. This phenomenon can be exploited to couple a surface acoustic wave to a transmon with a suitably shaped capacitance. The coupling strength between a transmon and a SAW cavity can be calculated by considering the charge q and the potential difference V_0 generated by a single phonon on the electrodes of the transmon. Let us first derive the potential difference V_0. The

zero-point mechanical motion associated to a single phonon inside a SAW cavity is:

$$U_0 = \sqrt{\frac{\hbar}{2\rho A_{\mathrm{c}} v_{\mathrm{e}}}}, \tag{3}$$

where $A = W \times L_{\mathrm{c}} = 95\,900\,\mu\mathrm{m}^2$ is the area of the acoustic cavity and $\rho = 2647\,\mathrm{kg/m^3}$ is the quartz mass density. From the zero-point mechanical motion, we can easily derive the value of the zero-point electric potential[2]:

$$\phi_0 \approx \frac{e_{\mathrm{pz}}}{\varepsilon} U_0 = \frac{e_{\mathrm{pz}}}{\varepsilon}\sqrt{\frac{\hbar}{2\rho A_{\mathrm{c}} v_{\mathrm{e}}}}, \tag{4}$$

where ε is the permittivity of the substrate and e_{pz} is a component of the quartz piezoelectric tensor which depends on the propagation direction (for ST-X quartz[3], $e_{\mathrm{pz}}/\varepsilon \approx 2.0\,\mathrm{V/nm}$). As mentioned earlier, the transmon capacitance responds in a more effective way to waves sharing the same periodicity of its structure. Hence, the electric potential ϕ_0 has to be scaled according to the following normalised array factor[3]:

$$A(f) = \left| \frac{\sin\left[N_{\mathrm{q}}\pi(f - f_0)/2f_0\right]}{N_{\mathrm{q}}\pi(f - f_0)/2f_0} \right|. \tag{5}$$

Note that for $f = f_0$, $A(f) = 1$. The potential difference is thus $V_0 = \phi_0 A(f)$. As regards the charge generated by the surface acoustic wave on the transmon electrodes, its value is given by:

$$\hat{q} = 2e\beta\hat{n} \tag{6}$$

where $2e$ is the charge of a Cooper pair, β is a prefactor, $|j\rangle$ is an eigenstate of the transmon and $\hat{n}$ is a quantum operator indicating the number of Cooper pairs in excess (or deficit) on the superconducting island. To calculate the coupling in the transmon eigenbasis, we need the matrix element[4]:

$$\langle j+1|\hat{q}|j\rangle = 2e\beta\langle j+1|\hat{n}|j\rangle \approx 2e\beta\sqrt{\frac{j+1}{2}}\left(\frac{E_{\mathrm{J}}(\phi)}{8E_{\mathrm{C}}}\right)^{1/4}. \tag{7}$$

For the first two levels of the transmon, we have $\langle 1|\hat{q}|0\rangle \approx e\beta\left(E_{\mathrm{J}}(\phi)/2E_{\mathrm{C}}\right)^{1/4}$. It remains to calculate the prefactor β. This parameter originates from the fact that not all of the charge generated by the surface acoustic wave will be localised on the transmon capacitance: part of it will be distributed on the gate capacitance C_2, on the junction capacitance C_{J} and strain capacitances C_s to ground planes and other metallic components of the chip. Hence:

$$\beta = \frac{C_{\mathrm{q}}}{C_2 + C_s + C_{\mathrm{J}} + C_{\mathrm{q}}} = \frac{C_{\mathrm{q}}}{C_\Sigma}. \tag{8}$$

where $C_{\mathrm{q}} = WN_{\mathrm{q}}\varepsilon/2$ is the capacitance of the qubit interdigitated structure. The coupling strength between a transmon and a SAW cavity can be written as follows:

$$\begin{aligned} \lambda(\phi, f) &= \frac{\langle 1|\hat{q}|0\rangle\, V_0}{h} \approx \frac{2e}{h}\beta\langle 1|\hat{n}|0\rangle\phi_0 A(f) = \\ &= \frac{e\beta}{h}\left(\frac{E_{\mathrm{J}}(\phi)}{2E_{\mathrm{C}}}\right)^{1/4}\phi_0 A(f), \end{aligned} \tag{9}$$

where the value of $E_{\mathrm{J}}(\phi)$ depends on the qubit frequency in the following way $E_{\mathrm{J}}(\phi) = [hf_{\mathrm{q}}(\phi) + E_{\mathrm{C}}]^2/8E_{\mathrm{C}}$. Substituting values related to our device and assuming that the frequency of the qubit is $f_{\mathrm{q}} = 2.52\,\mathrm{GHz}$ and approximating $\beta \approx 1$ and $A(f) \approx 1$, we have:

$$\begin{aligned} \lambda &\approx \frac{e}{h}\left(\frac{3.4\,[\mathrm{GHz}]}{2.0 \times 0.31\,[\mathrm{GHz}]}\right)^{1/4} 2\left[\frac{\mathrm{nV}}{\mathrm{m}}\right]\sqrt{\frac{\hbar}{2 \times 2647\,[\mathrm{kg/m^3}] \times 95\,900\,[\mu\mathrm{m}^2] \times 3140.6\,[\mathrm{m/s}]}} = \\ &= 6.0\,\mathrm{MHz}. \end{aligned} \tag{10}$$

This value agrees well with the experimental value extracted from our measurements.

Supplementary Note 3: Cryogenic setup and fabrication procedure

The device presented in this work has been characterised at cryogenic temperatures. The microchip has been bonded on a circuit board, placed inside a home-made oxygen-free copper sample holder

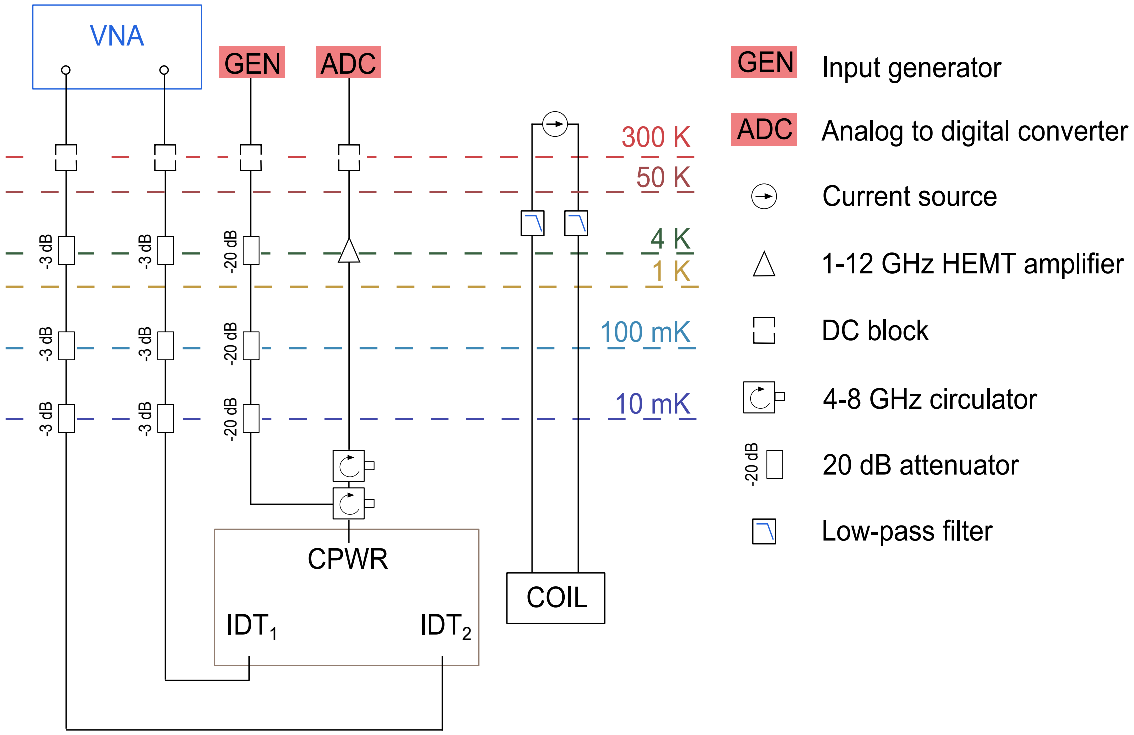

Supplementary Figure 4 | Cryogenic setup. Schematic of our experimental setup, showing the microwave control and measurement circuit connected to the sample, embedded in a dilution refrigerator.

and mechanically anchored to the $10\,\mathrm{mK}$ plate of a dilution refrigerator (Triton200, Oxford Instruments). The microwave line connecting the CPWR to the external instrumentation is highly attenuated by means of three $-20\,\mathrm{dBm}$ attenuators. The measured attenuation of this line is $-67\,\mathrm{dB}$. The reflected signal coming from the CPWR passes through two 4-8 GHz circulators and reaches a 1-12 GHz HEMT cold amplifier (see Supplementary Fig. 4). The signal is then downconverted and acquired using an analog-to-digital converter at room temperature. The lines connecting IDT_1 and IDT_2 to the VNA have an estimated overall attenuation of $-16\,\mathrm{dB}$.

We conclude this section presenting the fabrication procedure of our device. First of all, the ground planes, alignment marks and waveguides have been patterned with standard photolithographic techniques. These $100\,\mathrm{nm}$ thick metallic structures have been deposited with a home-made electron-beam evaporator. The SAWR and the qubit have been patterned together in a second electron-beam lithography step. The $200 \times 200\,\mathrm{nm}^2$ junctions have been fabricated with the usual Dolan bridge technique and with a double angle evaporation in the following way: firstly $30\,\mathrm{nm}$ of aluminium have been deposited, followed by an in-situ oxidation step and a second deposition of $60\,\mathrm{nm}$ of aluminium. The overall height of the qubit and SAWR is therefore $90\,\mathrm{nm}$.

Supplementary References

1. Manenti, R. *et al.* Surface acoustic wave resonators in the quantum regime. *Phys. Rev. B* **93**, 041411 (2016).

2. Schuetz, M. J. A. *et al.* Universal Quantum Transducers Based on Surface Acoustic Waves.

Phys. Rev. X **5**, 031031 (2015).

3. Morgan, D., *Surface Acoustic Wave Filters.* (Academic Press, Amsterdam, 2007).

4. Koch, J. *et al.* Charge-insensitive qubit design derived from the Cooper pair box. *Phys. Rev. A* **76**, 042319 (2007).
